# The association of ^18^F-deoxyglucose (FDG) uptake of PET with polymorphisms in the glucose transporter gene (*SLC2A1*) and hypoxia-related genes (*HIF1A*, *VEGFA*, *APEX1*) in non-small cell lung cancer. *SLC2A1 *polymorphisms and FDG-PET in NSCLC patients

**DOI:** 10.1186/1756-9966-29-69

**Published:** 2010-06-12

**Authors:** Seong-Jang Kim, Sang-Hyun Hwang, In Joo Kim, Min Ki Lee, Chang Hun Lee, Sang-Yull Lee, Eun Yup Lee

**Affiliations:** 1 Departments of Nuclear Medicine, Pusan National University Hospital, School of Medicine Pusan National University, Busan, Korea; 2 Departments of Laboratory Medicine, Pusan National University Hospital, School of Medicine Pusan National University, Busan, Korea; 3 Departments of Internal Medicine, Pusan National University Hospital, School of Medicine Pusan National University, Busan, Korea; 4 Departments of Pathology, Pusan National University Hospital, School of Medicine Pusan National University, Busan, Korea; 5 Department of Biochemistry, School of Medicine Pusan National University, Busan, Korea; 6 Medical Research Institute, Pusan National University Hospital, Busan, Korea

## Abstract

**Background:**

Positron emission tomography imaging of lung cancers with 2-[fluorine-18]-fluoro-2-deoxy-D-glucose is a non-invasive diagnostic, and prognostic tool that measures tumor metabolism. We have analyzed the effect of solute carrier family 2 (facilitated glucose transporter), member 1 polymorphisms on 2-[fluorine-18]-fluoro-2-deoxy-D-glucose-uptake with a combination of polymorphisms of hypoxia-inducible factor 1 alpha, apurinic/apyimidinic endonuclease, and vascular endothelial growth factor A in a hypoxia-related pathway.

**Methods:**

We investigated the association between solute carrier family 2 (facilitated glucose transporter), member 1 -2841A>T, hypoxia-inducible factor 1 alpha Pro582Ser, Ala588Thr, apurinic/apyimidinic endonuclease Asp148Glu, or vascular endothelial growth factor A +936C>T and 2-[fluorine-18]-fluoro-2-deoxy-D-glucose-uptake among 154 patients with non-small-cell lung cancer.

**Results:**

The solute carrier family 2 (facilitated glucose transporter), member 1 -2841A>T polymorphism was significantly associated with 2-[fluorine-18]-fluoro-2-deoxy-D-glucose-uptake in combination with the apurinic/apyimidinic endonuclease Asp148Glu (T>G) polymorphism in the squamous cell type of non-small-cell lung cancer. The solute carrier family 2 (facilitated glucose transporter), member 1 TT genotype had a higher maximum standardized uptake values than the AA + AT genotype when the apurinic/apyimidinic endonuclease genotype was TT (mean maximum standardized uptake values, 12.47 ± 1.33 versus 8.46 ± 2.90, respectively; *P *= 0.028). The mean maximum standardized uptake values were not statistically different with respect to vascular endothelial growth factor A and hypoxia-inducible factor 1 alpha polymorphisms.

**Conclusion:**

A glucose transporter gene polymorphism was shown to be statistically associated with glucose-uptake when the apurinic/apyimidinic endonuclease genotype is TT in patients with the squamous cell type of non-small-cell lung cancer. Our findings suggest that a newly developed tracer for positron emission tomography could be affected by genetic polymorphisms.

## Introduction

Positron emission tomography (PET) imaging of malignant tumors with 2-[fluorine-18]-fluoro-2-deoxy-D-glucose (FDG) as a tracer (FDG uptake depends on glucose uptake) is a non-invasive diagnostic, and prognostic tool that measures tumor metabolism. FDG-PET has been shown to have the ability to diagnose disease. Moreover, FDG-PET is used for treatment planning and is used to evaluate the response to therapy [1]. The *SLC2A1 *(also called glucose transporter type 1, *GLUT1*) gene is the primary glucose transporter gene in human lung cancer [2].

Hypoxia-inducible factor 1a (HIF-1a) controls oxygen delivery via angiogenesis and metabolic adaptation to hypoxia via glycolysis [3]. HIF-1a regulates *SLC2A1 *gene expression in cells that are subjected to hypoxic conditions [4]. Many cellular proteins interact with or are under the control of HIF1-a. HIF-1a overexpression and enhanced transcriptional activity are linked to tumor initiation and progression. Indeed, a large number of clinicopathologic studies have confirmed that unlike mature normal tissues, HIF-1a is overexpressed in the cytoplasm and nuclei of 40%-80% of human carcinomas, including lung, breast, head and neck, endometrial cancers, melanomas, and sarcomas [5,6]. Recently, Fu *et al*. [7] and Koukourakis *et al*. [8] showed that a *HIF1A *gene polymorphism affected HIF-1a protein expression.

The expression of the downstream *SLC2A1 *and vascular endothelial growth factor (*VEGF*) genes are regulated by a *HIF1A*-activated transcription pathway. *VEGFA *is the major mediator of angiogenesis and vascular permeability and transcription of this gene under hypoxic conditions depends on HIF-1a induction. A C>T polymorphism at position 936 in the 3' untranslated region of the *VEGFA *gene has been associated with the plasma levels of *VEGFA *[9]. The T variant, which is linked to lower *VEGFA *levels, has been associated with colon cancer [10] and low FDG uptake [11]. These findings suggest a potential role of the *VEGFA *936C>T polymorphism for the variability of FDG uptake in tumor tissues.

One important mammalian redox modulator is the bifunctional enzyme Redox factor-1 (Ref-1, also termed *APEX1*), that promotes transcriptional activation of HIF-1 or hypoxia inducible factor-like factor (HLF) by reducing C-terminal domain of HIF-1 or HLF [12], although the major role of this enzyme is DNA base excision repair [13]. Recently, APEX polymorphisms have been the focus of studies involving several different types of cancer, including colorectal [14], breast [15], and non-small cell lung cancer (NSCLC) [16]. These results suggested the involvement of *APEX1 *in the development of lung cancer.

The proteins encoded by *SLC2A1 *and *VEGFA *are under the control of *HIFA *gene expression. An effect of these gene polymorphisms on glucose uptake to modify FDG-uptake could be influenced by the interaction of proteins in a common pathway. In this study, we have determined the impact of *SLC2A1 *polymorphisms on FDG-uptake (maximum standardized uptake value [SUVmax]) using a pathway-based approach with a combination of *HIFA*, *APEX1*, and *VEGFA *gene polymorphisms that might influence glucose uptake.

## Materials and methods

### 1. Patient characteristics

Patients suspected of having lung cancer were prospectively recruited between October 2005 and October 2008. The inclusion criteria were as follows: (1) patients had a pathologically-confirmed diagnosis of NSCLC (2) and peripheral blood lymphocytes and FDG-PET images were available for analysis. Patients had a standard staging work-up that included fibroscopy, a chest and abdominal CT scan, brain MRI or CT imaging, and FDG-PET. One hundred fifty-four patients with NSCLC met the inclusion criteria with a median follow-up time of 7.5 months (range, 0.13 - 29.5 months). There were 62 deaths (40.3%) during the study period.

#### Single nucleotide polymorphism Selection

Single nucleotide polymorphisms (SNPs) were chosen for non-synonymous coding polymorphisms or for clinically-associated polymorphisms described in previous studies. The following SNPs were selected in this study: *SLC2A1 *-2841A>T (rs710218), *VEGFA*+936C>T (rs3025039) [NM_001025366.1:c.*237C>T], *APEX1 *Asp148Glu (T>G, rs1130409) [NM_001641.2:c.444T>G], *HIF1A *Pro582Ser (C>T, rs11549465) [NM_001530.2:c.1744C>T], and *HIF1A *Ala588Thr (G>A, rs11549467) [NM_001530.2:c.1762G>A].

#### Genotyping

The SNaPshot assay was performed according to the manufacturer's instructions (ABI PRISM SNaPShot Multiplex kit; Applied Biosystems, Foster City, CA, USA). Briefly, the genomic DNA flanking the SNP of interest was amplified with the use of a PCR reaction with forward and reverse primer pairs and standard PCR reagents. The 10 μL reaction volume contained 10 ng of genomic DNA, 0.5 pM of each oligonucleotide primer, 1 mL of 10× PCR buffer, 250 μM dNTP (2.5 mM each), and 0.25 units i-StarTaq DNA Polymerase (5 units/μL; iNtRON Biotechnology, Sungnam, Kyungki-Do, Korea). PCR reactions were carried out as follows: 10 min at 95°C for 1 cycle, and 35 cycles at 95°C for 30 s, followed by 1 extension cycle at 72°C for 10 min. After amplification, the PCR products were treated with 1 U each of shrimp alkaline phosphatase (SAP) and exonuclease I (Roche Diagnostics, Mannheim, Germany) at 37°C for 75 min and 72°C for 15 min to purify the amplified products. One μL of the purified amplification products was added to a SNaPshot Multiplex Ready reaction mixture containing 0.15 pmol of genotyping primer for a primer extension reaction. The primer extension reaction was carried out for 25 cycles of 96°C for 10 sec, 50°C for 5 sec, and 60°C for 30 sec. The reaction products were treated with 1 U of SAP at 37°C for 1 hr and 72°C for 15 min to remove excess fluorescent dye terminators. One μL of the final reaction samples containing the extension products was added to 9 μL of Hi-Di formamide (Applied Biosystems). The mixture was incubated at 95°C for 5 min, followed by 5 min on ice, then the mixture was analyzed by electrophoresis on an ABI Prism 3730xl DNA analyzer. Analysis was carried out using Genemapper software (version 3.0; Applied Biosystems). Table 1 shows the primer sets and Tm used for the SNaPshot assay.

**Table 1:** Primers for amplification and allele frequencies of hypoxia-related polymorphisms

Gene	SNP name (rs number)	Primer sequence		Tm
*VEGFA*	+936C>T (rs3025039)	Forward	CACTTTGGGTCCGGAGGG	
		Reverse	TGATTTAGCAGCAAGAAAAATAAA	55
		Genotyping	GGCGAATCCAATTCCAAGAGGGACC	
*HIF1A*	P582S (rs11549465)	Forward	TATATCCCAATGGATGATGACTT	
		Reverse	TTCTTGTATTTGAGTCTGCTGG	55
		Genotyping	GTTACGTTCCTTCGATCAGTTGTCA	
*HIF1A*	A588T (rs11549467)	Forward	AGATTTAGACTTGGAGATGTTAGCTC	
		Reverse	AATACTGTAACTGTGCTTTGAGGAC	55
		Genotyping	GTTGTCAYCATTAGAAAGCAGTTCC	
*SLC2A1*	-2841A>T (rs710218)	Forward	CCTTTGCACATGCTCTTC	
		Reverse	GCCACCTTGTCCTTCTCT	60
		Genotyping	CCTGTGGTACAAGTTCTGC	
*APEX1*	D148E (rs1130409)	Forward	CAGTGCCCACTCAAAGTT	
		Reverse	CTTGCGAAAGGCTTCAT	60
		Genotyping	GGCCTTCCTGATCATGCTCCTC	

#### F-18 FDG PET

F-18 FDG PET imaging was performed with a dedicated PET/CT scanner (Gemini; Philips, Milpitas, CA USA) that consisted of a dedicated germanium oxyorthosilicate full-ring PET scanner and a dual-slice helical CT scanner. The standard patient preparation included at least 8 hours of fasting and patients with a serum glucose level < 120 mg/dL before F-18 FDG administration. PET/CT imaging was performed 60 minutes after the injection of F-18 FDG. Sixty minutes after the administration of F-18 FDG, low-dose CT (30 mAs, 120 kV) covering the area from the base of the skull to the proximal thighs was performed for the purpose of attenuation correction and precise anatomic localization. Thereafter, an emission scan was conducted in the three-dimensional mode. The emission scan time per bed position was 3 minutes and 9 bed positions were acquired. PET data were obtained using a high-resolution whole body scanner with an axial field of view of 18 cm. The average total PET/CT examination time was 30 minutes. After scatter and decay correction, PET data were reconstructed iteratively with attenuation correction and were reoriented in axial, sagittal, and coronal slices. A row action maximum-likelihood algorithm was used for three-dimensional reconstruction.

#### Visual assessment and quantitative analysis

Experienced nuclear medicine physicians blinded to the results of other imaging modalities and the pathologic findings reviewed the F-18 FDG PET/CT scans. The medical records, including treatment regimens, other medical imaging modalities, and fine needle aspiration biopsies, were reviewed and analyzed. Two experienced nuclear medicine physicians independently reviewed the PET/CT images and any disagreement was resolved by consensus. To calculate the SUVmax, manually-defined circular regions of interest (ROI) were drawn on the attenuation-corrected emission images throughout the axial planes where a suspicious lesion could be delineated.

### Statistical analysis

The association between the mean SUVmax and clinicopathologic factors was analyzed using a two-tailed Pearson's chi-squared or Fisher's exact test as appropriate. Differences in groups for the mean SUVmax values were tested using one-way ANOVA or the t-test as appropriate. For the t-test, we assessed the equality of variance with Levene's test. Differences were considered significant when the *P *value was < 0.05. Statistical analysis and Kaplan-Meier curves were performed with SPSS (version 14.0; SPSS, Inc., Chicago, IL, USA).

## Results

### 1. Patient characteristics

The median patient age was 65 years (range, 28-84 years); 114 (74.0%) of the patients were men. The majority (83.1%) of patients had stage III or IV disease. Seventy-five of the patients (48.7%) had adenocarcninomas and 79 (51.3%) had squamous cell carcinomas. The clinicopathologic data are summarized in Table 2.

**Table 2:** Patient characteristics

		Adenocarcinoma	Squamous cell carcinoma
Age			
	Male	64.2 ± 8.5 (n = 41)	66.0 ± 8.1 (n = 73)
	Female	59.2 ± 10.8 (n = 34)	67.7 ± 10.0 (n = 6)
Smoking habit			
	Never	35 (46.7%)	7 (8.9%)
	Smoker	40 (53.3%)	72 (91.1%)
Stage			
	Stage I + II	14 (18.7%)	12 (15.2%)
	Stage III + IV	61 (81.3%)	67 (84.8%)
T stage			
	1	12 (16.0%)	4 (5.1%)
	2	2 (2.7%)	8 (10.1%)
	3	19 (25.3%)	43 (54.4%)
	4	42 (56.0%)	24 (30.4%)

### 2. Genotype information

The Hardy-Weinberg equilibrium was observed for all SNPs. The frequencies of the AA, AT, and TT genotypes of *SLC2A1 *-2841A>T were 51.7%, 37.7%, and 10.6%, respectively. Other genotype frequencies are listed in Table 3. Using the Haploview v. 4.0 software package, we constructed haplotypes of *HIF1A *Pro582Ser and Ala588Thr. *HIF1A *was nearly monomorphic and CCGG was most commonly observed with a frequency of 81.6%.

**Table 3:** Allele frequencies of *SLC2A1*, *VEGFA*, *APEX1*, and *HIF1A *polymorphisms

Target gene polymorphism (rs number)	Genotype	No. patients	(%)	Allele frequencies		Hardy-Weinberg equilibrium
***SLC2A1* -2841A>T**	AA	78	(51.7%)	A:T	0.705:0.295	0.2579
**(rs710218)**	AT	57	(37.7%)			
	TT	16	(10.6%)			
***VEGFA* +936C>T**	CC	102	(67.1%)	C:T	0.819:0.181	0.2579
**(rs3025039)**	CT	45	(29.6%)			
	TT	5	(3.3%)			
***APEX1 *Asp148Glu**	TT	55	(36.4%)	T:G	0.589:0.411	0.3929
**(rs1130409)**	TG	68	(45.0%)			
	GG	28	(18.5%)			
***HIF1A *Pro582Ser**	CC	139	(90.8%)	C:T	0.954:0.046	0.5541
**(rs11549465)**	CT	14	(9.2%)			
	TT	0	(0.0%)			
***HIF1A *Ala588Thr**	GG	137	(90.1%)	G:A	0.951:0.049	0.5219
**(rs11549467)**	GA	15	(9.9%)			
	AA	0	(0.0%)			

### 3. Association of SNPs with the mean SUVmax

No statistical differences were observed between the SNPs and the mean SUVmax when the patients were not stratified. We classified the patients into two groups according to the histologic cell type (adenocarcinoma and squamous cell carcinoma). There were no significant differences between the SNPs and the mean SUVmax in patients with adenocarcinomas. In patients with squamous cell carcinomas, the mean SUVmax of the *SLC2A1 *TT and AA + AT genotypes (recessive model) were 10.64 ± 2.26 and 9.07 ± 2.79, respectively, with no statistical significance (*P *= 0.130, Table 4).

**Table 4:** Association between gene polymorphisms of hypoxia-related genes and the mean SUVmax in patients with squamous cell carcinoma (n = 78)

Genes	Genotype	SUVmax	P*	Domonant model	SUVmax	***P***^†^	Recessive mode	SUVmax	*P*^†^
***SLC2A1* -2841A>T**	AA (n = 41)	9.40 ± 2.63	0.155	AA	9.40 ± 2.63	0.565	AA + AT	9.07 ± 2.79	*0.130*
	AT (n = 29)	8.60 ± 2.98		AT + TT	9.04 ± 2.94		TT	10.64 ± 2.26	
	TT (n = 8)	10.64 ± 2.26							
***VEGFA *+936C>T**	CC (n = 54)	9.29 ± 2.66	0.816	CC	9.29 ± 2.66	0.774	CC + CT	9.20 ± 2.80	*0.663*
	CT (n = 20)	8.95 ± 3.23		CT + TT	9.10 ± 3.06		TT	9.83 ± 2.25	
	TT (n = 4)	9.83 ± 2.25							
***APEX1 *Asp148Glu**	TT (n = 28)	8.89 ± 3.04	0.522	TT	8.89 ± 3.04	0.412	TT + TG	9.30 ± 2.90	*0.672*
	TG (n = 34)	9.64 ± 2.79		TG + GG	9.43 ± 2.62		GG	8.97 ± 2.23	
	GG (n = 16)	8.97 ± 2.23							
***HIF1A *Pro582Ser**	CC (n = 69)	9.32 ± 2.84	0.671						
	CT (n = 10)	8.92 ± 2.35							
***HIF1A *Ala588Thr**	GG (n = 68)	9.18 ± 2.74	0.664						
	GA (n = 10)	9.59 ± 3.11							

*ANOVA † t-test

### 4. Association of SNPs on the mean SUVmax in squamous cell carcinomas

We analyzed subgroups according to the combinations of *SLC2A1*, *VEGFA*, *APEX1*, and *HIF1A *polymorphisms in patients with squamous cell carcinomas. The *SLC2A1 *-2841A>T polymorphism was significantly associated with the mean SUVmax in the recessive model of *SLC2A1 *-2841A>T in combination with the *APEX1 *polymorphism (Table 5). For the TT genotype of *APEX1*, the *SLC2A1 *TT genotype had a higher SUVmax than the AA + AT genotype (12.47 ± 1.33 versus 8.46 ± 2.90, respectively; *P *= 0.028, Table 5). The other combinations of SLC2A1, *VEGFA*, and *HIF1A *polymorphisms were not associated with the mean SUVmax.

**Table 5:** Association between the *SLC2A1 *-2841A>T gene polymorphism and the mean SUVmax in patients with squamous cell carcinoma according to the *APEX1 *genotype

*APEX1* genotype	Gene	genotype	SUVmax	P*	Dominant model	SUVmax	***P***^†^	Recessive mode	SUVmax	*P*^†^
TT	*SLC2A1 *-2841A>T	AA (n = 13)	8.68 ± 2.40	0.086	AA	8.68 ± 2.40	0.742	AA + AT	8.46 ± 2.90	0.028
		AT (n = 12)	8.22 ± 3.47		AT + TT	9.07 ± 3.58		TT	12.47 ± 1.33	
		TT (n = 3)	12.47 ± 1.33							
TG	*SLC2A1 *-2841A>T	AA (n = 20)	9.72 ± 3.00	0.984	AA	9.72 ± 3.00	0.857	AA + AT	9.66 ± 2.93	0.932
		AT (n = 9)	9.53 ± 2.94		AT + TT	9.54 ± 2.56		TT	9.54 ± 2.00	
		TT (n = 5)	9.54 ± 2.01							
GG	*SLC2A1 *-2841A>T	AA (n = 8)	9.81 ± 1.97		AA	9.81 ± 1.97	0.134	AA + AT	8.97 ± 2.23	
		AT (n = 8)	8.13 ± 2.26		AT + TT	8.13 ± 2.26		TT		
		TT (n = 0)								

*ANOVA

† t-test

## Discussion

Although there have been several reports that have described an association between hypoxia-related genes and SUVmax in patients with lung cancer [17,18], this is the first study that has evaluated the impact of *SLC2A1 *gene polymorphisms on FDG-uptake in conjunction with the HIF-1a-activated transcription pathway in patients with NSCLC. With this pathway-based approach, we have demonstrated that *SLC2A1 *TT is statistically associated with a high FDG-uptake in combination with the TT genotype of *APEX1 *in patients with the squamous cell type of NSCLC. Interestingly, the *SLC2A1 *TT and *APEX1 *TT genotypes are risk alleles associated with the clinical outcome of several diseases, including diabetes and other malignancies [19,20].

FDG-uptake of PET, expressed as the SUVmax, is largely dependent on glucose metabolism in lung cancer. *SLC2A1 *is the primary glucose transporter of glucose metabolism and overexpression of *SLC2A1 *has an important role in the survival and rapid growth of cancer cells in a suboptimal environment [2]. High FDG uptake is associated with reduced overall survival and disease-free survival of patients [21].

*SLC2A1 *protein expression was shown to differ based on the histologic type in patients with NSCLC. The expression of *SLC2A1 *in squamous cell carcinomas was higher than adenocarcinomas[2]. Growth rate has been reported to be faster in squamous cell carcinomas, but slower in adenocarcinomas [22], and lung tumor growth correlates with glucose metabolism [23]. In our study, the significance of *SLC2A1 *gene polymorphisms on FDG-uptake was consistently observed for squamous cell carcinomas, but not for adenocarcinomas.

The functional effect of the *SLC2A1 *-2841A>T polymorphism has not been completely characterized. A hypoxia response element (HRE) is located 400 bp downstream from the A-2841T site. The close proximity of the polymorphism to the HRE may modify the binding affinity of HIF-1 and may alter the efficiency of the promoter and expression of *SLC2A1 *[19]. The effect of the *SLC2A1 *polymorphism could be due to causative or linkage disequilibrium.

Although the XbaI polymorphism of *SLC2A1 *is a well-known polymorphism in diabetes, the association between diabetic nephropathy and the XbaI polymorphism in the *SLC2A1 *gene has been controversial in several case-control studies [24-26]. Furthermore, the polymorphic XbaI site is located on the second intron of the *SLC2A1 *gene. The allele cannot possibly cause changes in the protein sequence, and thus no change would be expected in *SLC2A1 *expression. Therefore, we did not evaluate the XbaI polymorphism of *SLC2A1*.

*APEX1 *promotes transcriptional activation of HIF-1 and HLF [12]. Reduced APEX1 protein expression demonstrated a reduction in tumor volume and FDG uptake, indicating that *APEX1 *affects glucose metabolism and cellular proliferation [27]. Homozygosity (TT genotype) for the *APEX1 *Asp148Glu variant genotype was significantly associated with a poorer overall survival [20].

Based on the observation that the statistical significance of a *SLC2A1 *gene polymorphism was clearly identified in combination with an *APEX1 *gene polymorphism, we reasoned that the clinical impact of a *SLC2A1 *gene polymorphism on FDG-uptake might be minimal in late stage NSCLC. The significant effect of the *APEX1 *TT genotype on the mean SUVmax with a *SLC2A1 *gene polymorphism in this study suggests a role for the *APEX1 *Asp148Glu polymorphism in FDG-uptake. However, an additional functional study for the effect of *APEX1 *gene polymorphisms on FDG-uptake at the cellular level should be performed.

We did not analyze *SLC2A1 *polymorphisms based on the genotype of *HIF1A *due to the nearly monomorphic status of *HIF1A *(the frequencies of *HIF1A *minor alleles were < 1%). These genotype frequencies were very similar to frequencies reported in a previous study by Kuwai *et al*. [28]. Kuwai and colleagues reported a CT polymorphism in 11%, but an absence of TT in the Japanese population. Moreover, despite the association of *HIF1A *polymorphisms with HIF-1a expression, there was no association of polymorphisms with the expression of the down-stream proteins encoded by *SLC2A1 *and *VEGFA *[8].

*VEGFA *is the major mediator of angiogenesis and vascular permeability. Transcription of *VEGFA *under hypoxic conditions depends on HIF-1a induction. Although FDG-uptake has been correlated significantly with *VEGFA *expression in patients with NSCLC [18], we did not observe an effect of the *VEGFA*+936C>T polymorphism on FDG-uptake. An association between the *VEGFA*+936C>T polymorphism and FDG-uptake has been rarely reported for patients with NSCLC. Wolf *et al*. [11] reported that the *VEGFA*+936C>T polymorphism is associated with FDG-uptake in breast cancer patients. The FDG-uptake data in the study by Wolf *et al*. [11] was expressed as categorical data (low, medium, and high uptake) and not as a SUVmax, as in the present study; thus, we cannot directly compare the values of SUVmax obtained in the present study. Another possible explanation was a difference in the study population. The population in the study by Wolf *et al*. [11] was breast cancer patients, while the study population in the present study was lung cancer patients. Recently, several functional SNPs of *VEFGA *have been identified that are associated with survival in patients with early stage NSCLC [29,30]. Well-documented functional SNPs, such as *VEGFA *+405G>C and -460T>C, should be evaluated to identify the association between *VEGFA *gene polymorphisms and FDG-uptake.

There were several limitations to this study. We did not evaluate the association between hypoxia-related gene polymorphisms and FDG-uptake in patients with early stage NSCLC. Although the *SLC2A1 *-2841A>T polymorphism in combination with the *APEX1 *Asp148Glu polymorphism was associated with FDG uptake in this study, this result was based on a statistical comparison rather than a functional study. Another limitation was the potential effect of unknown SNPs of hypoxia-related genes on FDG-uptake, as we only analyzed documented-functional SNPs. Thus, additional investigations of polymorphisms in entire hypoxia-induced pathway on FDG-uptake are needed.

In summary, the *SLC2A1 *-2841A>T polymorphism was associated with FDG-uptake in combination with the *APEX1 *TT genotype in patients with squamous cell carcinoma. Our findings suggest that a newly developed tracer for PET could be affected by genetic polymorphisms. However, further studies are required to validate these results.

## Competing interests

The authors declare that they have no competing interests.

## Authors' contributions

KSJ performed the molecular genetic studies and drafted the manuscript. KIJ participated in preparation of the manuscript. LMK and LCH participated in the design of the study and LSY performed the statistical analyses. LEY and HSH conceived the study, and participated in its design and coordination and helped to draft the manuscript. All authors read and approved the final manuscript.
